# PALSAR 50 m Mosaic Data Based National Level Biomass Estimation in Cambodia for Implementation of REDD+ Mechanism

**DOI:** 10.1371/journal.pone.0074807

**Published:** 2013-10-07

**Authors:** Ram Avtar, Rikie Suzuki, Wataru Takeuchi, Haruo Sawada

**Affiliations:** 1 Institute of Industrial Science, The University of Tokyo, Meguro-Ku, Tokyo, Japan; 2 Research Institute for Global Change, Japan Agency for Marine-Earth Science and Technology (JAMSTEC), Yokohama, Japan; 3 United Nations University Institute for Sustainability and Peace (UNU-ISP), Tokyo, Japan; DOE Pacific Northwest National Laboratory, United States of America

## Abstract

Tropical countries like Cambodia require information about forest biomass for successful implementation of climate change mitigation mechanism related to Reducing Emissions from Deforestation and forest Degradation (REDD+). This study investigated the potential of Phased Array-type L-band Synthetic Aperture Radar Fine Beam Dual (PALSAR FBD) 50 m mosaic data to estimate Above Ground Biomass (AGB) in Cambodia. AGB was estimated using a bottom-up approach based on field measured biomass and backscattering (σ^o^) properties of PALSAR data. The relationship between the PALSAR σ^o^ HV and HH/HV with field measured biomass was strong with R^2^ = 0.67 and 0.56, respectively. PALSAR estimated AGB show good results in deciduous forests because of less saturation as compared to dense evergreen forests. The validation results showed a high coefficient of determination R^2^ = 0.61 with RMSE  = 21 Mg/ha using values up to 200 Mg/ha biomass. There were some uncertainties because of the uncertainty in the field based measurement and saturation of PALSAR data. AGB map of Cambodian forests could be useful for the implementation of forest management practices for REDD+ assessment and policies implementation at the national level.

## Introduction

Forests play an important role in global carbon cycling, as they are potential carbon sinks and sources for atmospheric CO_2_ [1], [2]. Tropical forests store about 40% of the terrestrial carbon [3]. According to the FRA (2010) [4], the net change in global forest area was estimated to be −5.2 million ha per year for 2001–2010 and −8.3 million ha per year for 1990–2000. The Intergovernmental Panel on Climate Change (IPCC) has pointed out that reducing and/or preventing deforestation is the best possible mitigation option for climate change. Adopting afforestation and reforestation with the Clean Development Mechanism (CDM) under the Kyoto Protocol is not enough to mitigate climate change because deforestation releases more Green House Gases (GHGs) than afforestation and reforestation absorption. Forest conservation is only one of many possible options by which permanent land-use change may be avoided [5]. Reducing Emissions from Deforestation and forest Degradation (REDD+) mechanism avoids emissions of carbon into the atmosphere by conserving existing carbon stocks. The basic idea of REDD+ is to reward individuals, communities, projects and countries that reduce GHG emissions from deforestation [6]. REDD+ can promote a range of sustainability goals such as climate change mitigation, biodiversity conservation, sustainable use of forest and forest products, better livelihood for local peoples etc. [7]. Implementation of REDD+ mechanism require effective biomass and deforestation monitoring systems that could provide consistent results with reproducibility, and can be implemented at the national level [8]. Moreover, forest biomass information is useful for REDD+ carbon accounting and trading carbon credits [9]. Forest biomass information is also useful to understand efficiently the global carbon cycle and ecosystem processes, as well as to know how carbon stocks vary in relation to environmental conditions and human land use activities [10]. Forest carbon pools consist of trunks, branches, leaves, litter, dead wood, roots and soil carbon. However, most studies have focused on above ground biomass (AGB) because this is relatively large pool and other carbon pools can be calculated with simple equations [11].

There are various methodologies for biomass estimation but no current methodology presents a clear view on how carbon pools and their fluxes should be reported and what the accuracy and uncertainty of biomass monitoring might be. Therefore biomass mapping has become an urgent need to assess and produce data on forest carbon stocks and the change in these stocks at a national level [12]. A recent biomass map by Saatchi et al., (2011) [13] shows uncertainties of about 30–50% in Indo-China countries. Estimation of tropical forests biomass has been studied both with the optical and Synthetic Aperture Radar (SAR) data. Table 1 summarizes some previous studies related to forest biomass estimation.

**Table 1:** Previous studies related to forest biomass estimation.

No.	Authors	Study Area	Methodology	Data used
1	Sader et al., 1989 [14]	Luquillo Mountains, Puerto Rico	Normalized Difference Vegetation Index (NDVI)	Landsat MSS and TM, Simulator, airborne multispectral scanner
2	Beaudoin et al., 1994 [15]	Landes Forest, France	Adapted theoretical model	P-band SAR airborne
3	Rauste et al., 1994 [16]	Freiburg, south-east Germany; Ruotsinkyla, Finland	Linear regression analysis	AIRSAR C,L,P band
4	Brown et al., 1995 [17]	Rondonia State, Southwestern Brazilian Amazon	Allometric equation based on destructive sampling approach	Field measurement
5	Imhoff, 1995 [18]	Hawaii Volcanoes National Park	Multi polarization (HH, HV, VV) radar backscatter (σ) and polynomial regression model	NASA/JPL Airsar data with C, L, and P band
6	Harrell et al., 1997 [19]	South – eastern USA	Multiple regression analysis	SIR-C
7	Luckman et al., 1998 [20]	Tapajos, Para state and Manaus, Amazonas state, Brazil	Forest backscatter model	JERS-1 SAR L band
8	Steininger, 2000 [21]	Bolivia and Brazil	TM band 3,4,5 validated with allometric equation	Landsat TM
9	Austin et al., 2003 [22]	New South Wales, Australia	Linear regression analysis	JERS-1 SAR L band
10	Santos et al., 2003 [23]	Tapajos River region, Para state, Brazil	Regression models (logarithmic and polynomial function)	AeS-1 SAR P- band
11	Foody et al., 2003 [24]	Manaus (Brazil), Danum Valey (Malaysia) and Khun Khong (Thailand)	vegetation indices, complex band ratios complemented with multi-linear regression and neural networks method	Landsat TM
12	Lu, 2005 [25]	Eastern Brazilian Amazon: Altamira, Pedras, and Bragantina	LandsatTM bands, vegetation indices, band ratios, image transform (e.g. principal component analysis, Tasseled cap)	Landsat TM
13	Kuplich et al., 2005 [26]	Manaus and Tapajos forests, Brazil	Radar backscatter (σ) and GLCM texture based allometric equations	JERS-1 SAR image with L band
14	Watanabe et al., 2006 [27]	Temperate Coniferous forests	Multi-linear regression	PALSAR
15	Sales et al., 2007 [28]	Rondonia State, Southwestern Brazil	Stem volume – AGB equation and kriging method	Field data (RADAMBRASIL database)
16	Hajnsek et al., 2009 [29]	Mawas and Sungai Wain, Kalimantan, Indonesia	RVoG model and inversion of dual-polarization	Airborne multi-band (C, L, P, X band) and multi-polarization (PolInSAR)
17	Mitchard et al., 2009 [30]	Africa	Regression modelling	PALSAR
18	Lucas et al., 2010 [31]	Queensland, Australia	Regression modelling	PALSAR
19	Sun et al., 2011 [32]	Boreal forests of Howland, Maine (US)	Multi-linear regression analysis	LVIS and PALSAR
20	Sandberg et al., 2011 [33]	Hemiboreal forest, Sweden	Regression modelling	L-band and P-band SAR data
21	Saatchi et al., 2011 [13]	Tropical forests	Regression modelling	GLAS, MODIS, SRTM and QSCAT
22	Englhart et al., 2011 [34]	Tropical forest on Central Kalimantan, Indonesia	Regression modelling	TerraSAR-X and PALSAR
23	Mitchard et al., 2011 [35]	Central Africa (central Cameroon)	Regression modelling	PALSAR
24	Cartus et al., 2012 [36]	Northeastern United States	Water-Cloud model	PALSAR
25	Mutanga et al., 2012 [37]	South Africa	Regression modelling	WorldView-2
26	Carreiras et al., 2012 [38]	Guinea-Bissau (West Africa)	Regression modelling	PALSAR
27	Hame et al., 2013 [39]	Laos	Regression modelling and probability method	PALSAR and AVNIR-2
28	Suzuki et al., 2013 [40]	Boreal forests in Alaska	Regression modelling	PALSAR

The most accurate way of calculating biomass is destructive sampling and forest inventory data using allometric equations. However, these traditional techniques are often time consuming, labor intensive, difficult to implement, especially in remote areas, and they cannot provide the spatial distribution of biomass in large areas. Moreover, this method cannot provide historical information about the forest if no forest inventory data exists [25], [41]. Therefore, remote sensing data supplemented with forest inventory data can provide cheap and fast estimation as well as historical information about forest biomass. Most of the remote sensing techniques are based on optical and synthetic aperture radar (SAR) systems. The disadvantages of optical sensors are not related to plant structural parameters, availability of cloud-free images in tropical countries, and a low saturation level for spectral bands and various other vegetation indices [42]. Therefore, dependency on SAR data for biomass estimation has increased because SAR can provide data without the limitations of clouds and solar illumination. The high penetration capability of SAR allows more information extraction about plants structural parameters for improved biomass estimation [43], [44], [45].

The successful launch of the Advanced Land Observing Satellite's (ALOS) PALSAR in 2006 has increased the potential to use radar to measure biomass, as this is the first long-wavelength (L-band, 23-cm wavelength) SAR satellite sensor to have the capability of collecting single, dual, full and Scan-SAR mode with cross-polarized (HV, horizontal-transmit, vertical receive) and co-polarized (HH, horizontal-transmit, horizontal receive; VV, vertical-transmit, vertical receive) data. The HV polarization is useful because it interacts with trees and produces a strong response [35]. Various studies have analyzed the retrieval of AGB using radar data in tropical regions [30], [46], [47]. These methods are mostly based on empirical or semi-empirical relationship between radar backscatter and ground based data. Longer wavelengths SAR have proven to be more useful because of the increased backscatter range with changing biomass [48], [49], [50], [43]. These biomass estimations are valid up to a certain threshold where saturation occurs [51], [30]. Mitchard et al., 2009 [30] predicted above ground biomass in tropical Savanna forest of Africa with a saturation of PALSAR backscatter around 150 Mg/ha which is more than 100 Mg/ha predicted by Watanabe et al., 2006 [27]. Recently, Suzuki et al., 2013 [40] has also used PALSAR data to estimate biomass of boreal forests of Alaska and no obvious saturation was found up to 120 Mg/ha. In general, SAR saturation levels depend on the frequency of SAR systems as well as forest structure. The sensitivity of SAR decreases with the increase of biomass in dense forests [18], [52]. Most of the recent studies are focused on use of L-band SAR data (PALSAR) to estimate biomass because of high penetration capability [35], [36], [38]. However, there is no comprehensive study that uses PALSAR 50 m mosaic data to generate a national level biomass map. Therefore this study was carried out to estimate national level biomass based on a bottom-up approach to support REDD+ mechanism in Cambodia.

## Study area

Cambodia is located in Southeast Asia between 10°–15°N latitude and 102°–108°E longitude, covering about 181,037 km^2^ of area. Cambodia shares its border with Vietnam to the east, Thailand to the west, Lao P.D.R. to the north and Gulf of Thailand to the south. Cambodia is a country covered mainly by extensive plain lands and the Tonle Sap (Great Lake), which crosses the country from the north to the south. Topographically Cambodia is divided into two parts: (i) the central low lying or the central plains and (ii) the mountainous ranges. Central plains, consisting mainly of the alluvial plain of Mekong River and the Tonle Sap, cover about three quarters of the country's area [53].

**Figure 1:**
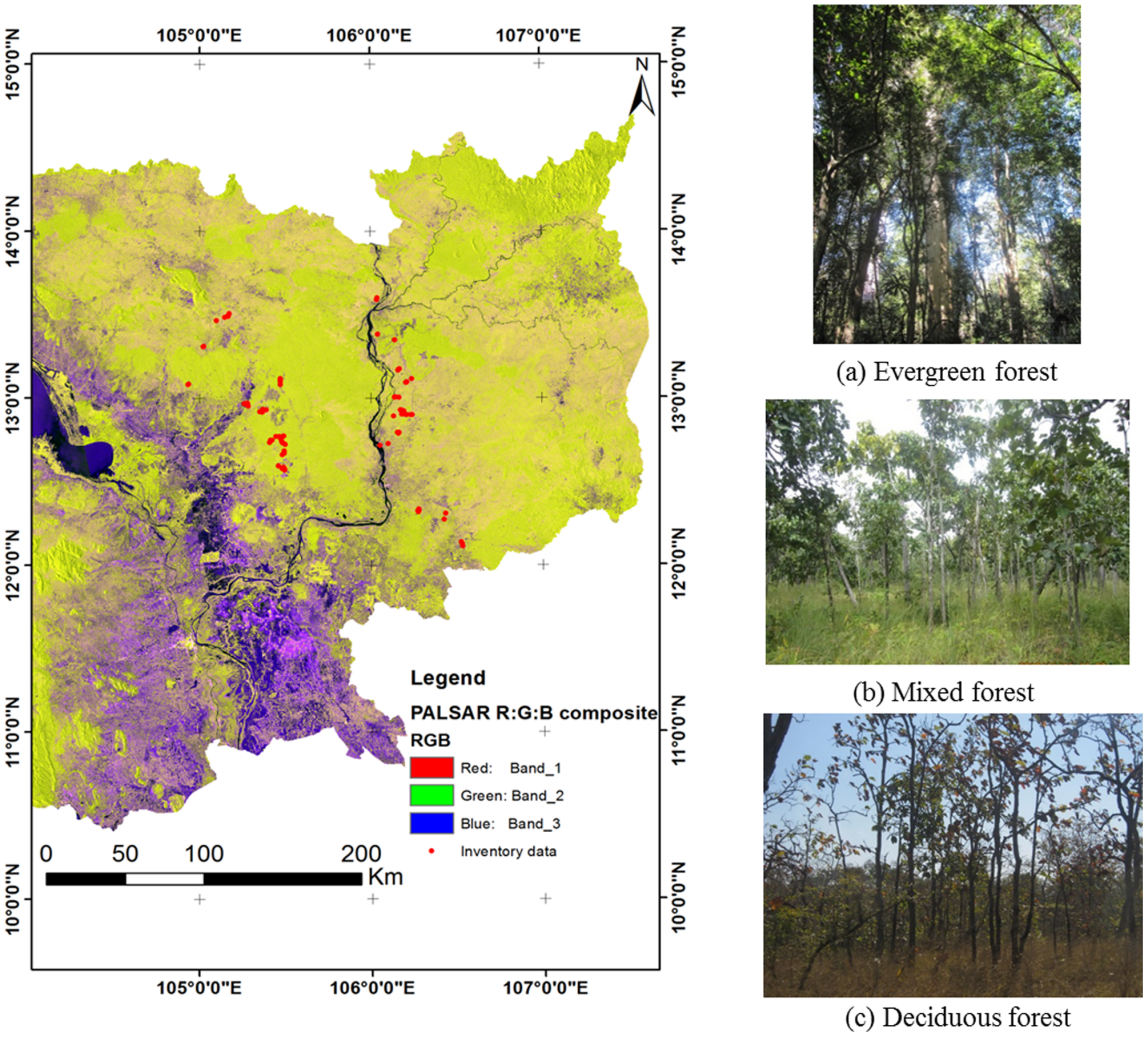
ALOS/PALSAR 50 m mosaic 2009, (Red: HH, Green: HV, Blue: HH/HV) data and locations of the inventory data in different forest types (a) evergreen (b) mixed and (C) deciduous forests of Cambodia.

Cambodia is a tropical country with two distinct seasons: the dry season from November to April and the rainy season from May to October. The mean annual precipitation depends on the region and ranges from 100 to 300 cm [54], [55], [56]. The heaviest rainfall, over 300 cm per year, occurs along the western coastal lowland area. Relative humidity ranges from 65–70% in January and February to 85–90% in August and September. Cambodia's average temperature ranges from 20° to 35°C. April is the warmest month, when the temperature can rise above 38°C and January is the coldest with temperature around 22°C [53]. Recent FRA (2010) data shows that Cambodia has the highest deforestation rate among Indo-China countries [4]. Logging activities, population growth, urbanization, and agricultural expansion have been the primary reason for Cambodia's forest loss [53], [57]. Cambodia signed United Nations REDD+ mechanism in 2009, therefore the study of forest biomass is necessary for REDD+ implementation. Figure 1 shows a R:G:B colour composite of PALSAR 50 m FBD data.

## Methodology

### a. Field data

Forest inventory data was collected with the help of Forestry Administration (FA) of Cambodia. We collaborated with FA to collect data in November 2010 and January 2011 with plot sizes of 30 m×60 m. Square plot design was used to facilitate pixel sampling based on satellite data to reduce position error. A systematic random sampling design was applied for the purpose of field data collection based on forest types with relatively homogeneous ecological conditions (i.e. topography, slope, distance from water source, soil types). Forest inventory parameters (Diameter at breast height (DBH), tree height, species, tree density and forest types) were collected from seventy nine plots. The tree diameter was measured at 1.3 m height above the ground using DBH tape with 1cm accuracy. The tree height was measured with the vertex hypsometer for all open trees with good visibility of the top and was estimated when it was not possible to see the top of the tree. Most sampling plots were selected in the plain area to minimize topographic effects of SAR data. The sampling plots were located using GPS (Garmin 62CSx). A total of seventy nine plots data were analyzed. Fifty one plots were used for the MLR model development and 23 plots were used for model validation. Five plots were excluded from the analysis because the location of the plots was near to the road as well as some degradation activity. The AGB in kg for each tree was calculated using the allometric equation as derived by Kiyono et al., (2010) [54]. Basal area of the plot was also calculated based on the DBH and tree density. Basal area (m^2^/ha) is defined as the cross-sectional area of all the trees in the plot divided by the area of the plot.

We used Kiyono et al., (2010) [54] allometric equations because Anglesen (2008) [6] has noticed that country specific allometric equation is better suited than using a global allometric equation. Kiyono et al., (2010) [54] method is originally designed for Cambodian forest and therefore there are less uncertainties as compared to other allometric equation. We also compared the Kiyono et al., (2010) allometric equation based biomass estimation with the Brown (1997) [58] and Kenzo et al., (2009) [59] allometric equations (Table 2) based biomass estimations. We found that the Brown (1997) [58] allometric equation based biomass estimation showed overestimation and Kenzo et al., (2009) [59] showed underestimation. The biomass value obtained from each tree with the Kiyono et al., (2010) [54] allometric equation were summed and normalized by the area of the plots to produce the AGB in Mg/ha. In this biomass estimation, we have only considered trees with ≥10 cm DBH, because they likely represent most of the woody mass of the plots.

**Table 2:** Allometric Equations.

No.	Author	Allometric equation
1	Kiyono et al., 2010 [54]	Leaf biomass (kg) = 173*(BA^0.938^) Branch biomass (kg) = 0.217*(BA^1.26^)*(D^1.48^) Stem biomass (kg) = 2.69*(BA^1.29^)*(D^1.35^) BA is basal area and D is stem density
2	Kenzo et al., 2009 [59]	Leaf biomass (kg) = 0.0442*(DBH^1.67^) Branch biomass (kg) = 0.0124*(DBH^2.48^) Stem biomass (kg) = 0.0822*(DBH^2.48^)
3	Brown et al., 1997 [58]	Biomass (kg) = (42.69–12.8*DBH+1.242*(DBH^2^))

### b. Satellite data

**Figure 2:**
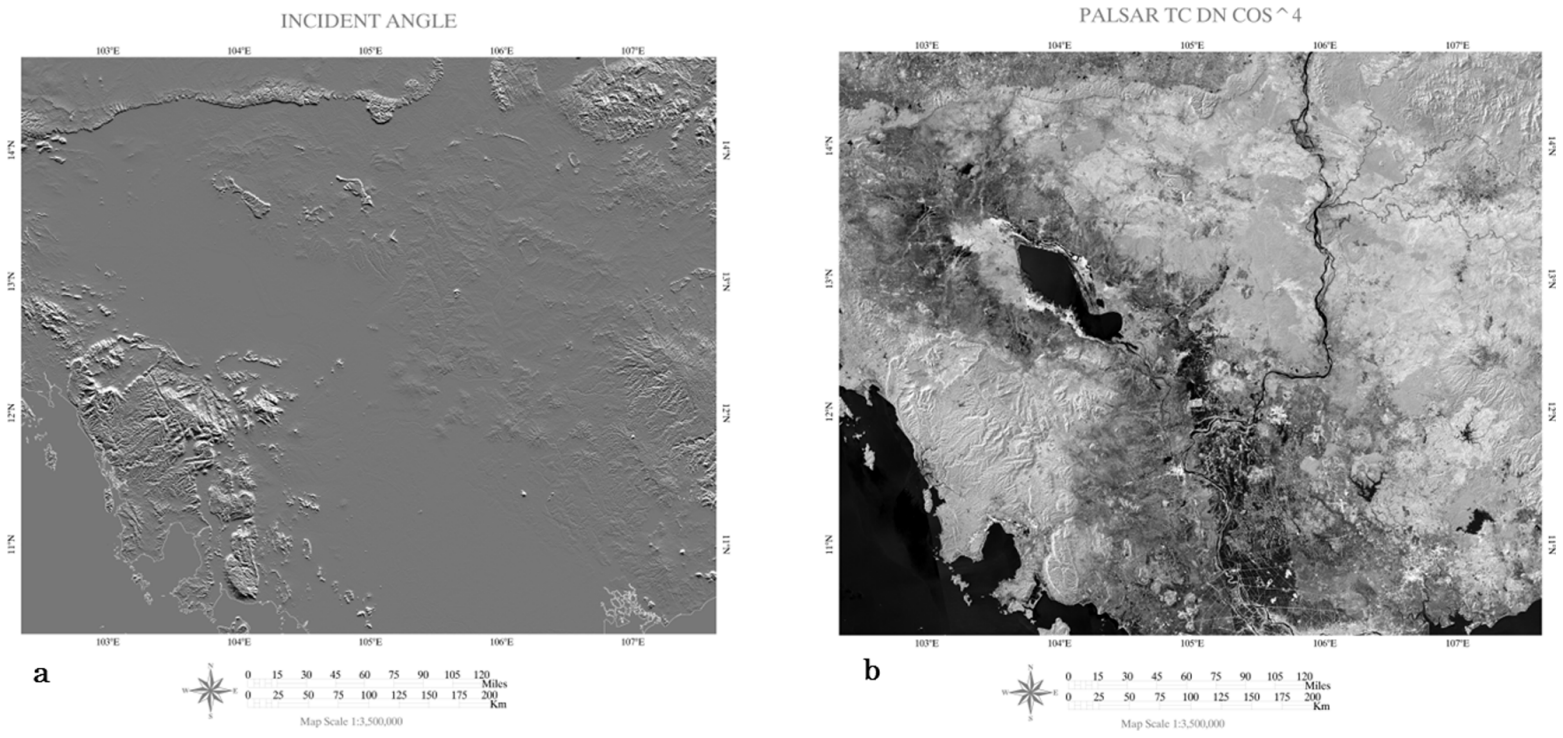
Incident angle based on slope and aspect image of SRTM-DEM data (a) and PALSAR terrain corrected image (b).



Land use/land cover map based on ASTER 2005 data [60], SRTM-DEM data, Landsat ETM+2009, 2010 data were used for selection of sampling sites. PALSAR FBD 50 m mosaic data was downloaded from Japan Aerospace Exploration Agency (JAXA). We have used dual polarization PALSAR data with HH and HV polarization. We have created R:G:B color composite image (HH: red, HV: green, and HH/HV: blue) (figure 1). The processing of PALSAR data was started with the terrain corrections using Shimada 2010 [61] methodology to minimize the topographic effects of PALSAR in mountainous areas. The PALSAR 50 m mosaic data were ortho-rectified using the SRTM DEM 90 m to correct the topography. The SRTM DEM with 90 m pixels were resampled to 50 m using bi-linear interpolation. Raster grids of resampled data was aligned with PALSAR 50 m mosaic data to minimize location error. Later on incidence angle was calculated based on slope and aspect of SRTM data [62]. Figure 2a and 2b shows the incident angle image based on slope and aspect of STRM data and terrain corrected PALSAR data respectively. Slope correction results shows that terrain correction methodology was not effective in high sleep mountainous area as compared to low mountainous areas. The digital number (DN) of PALSAR data was converted to the normalized radar cross section (NRSC or σ^o^) using the following equation (1) [63].

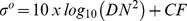
(1)where, σ^o^ is backscattering coefficient and CF is the calibration factor and its value for PALSAR dual polarimetric data is −83. We have not considered the climatic conditions of PALSAR 50 m mosaic data because different scenes were acquired on different date to make a mosaic.

### c. Statistical Analysis

**Figure 3:**
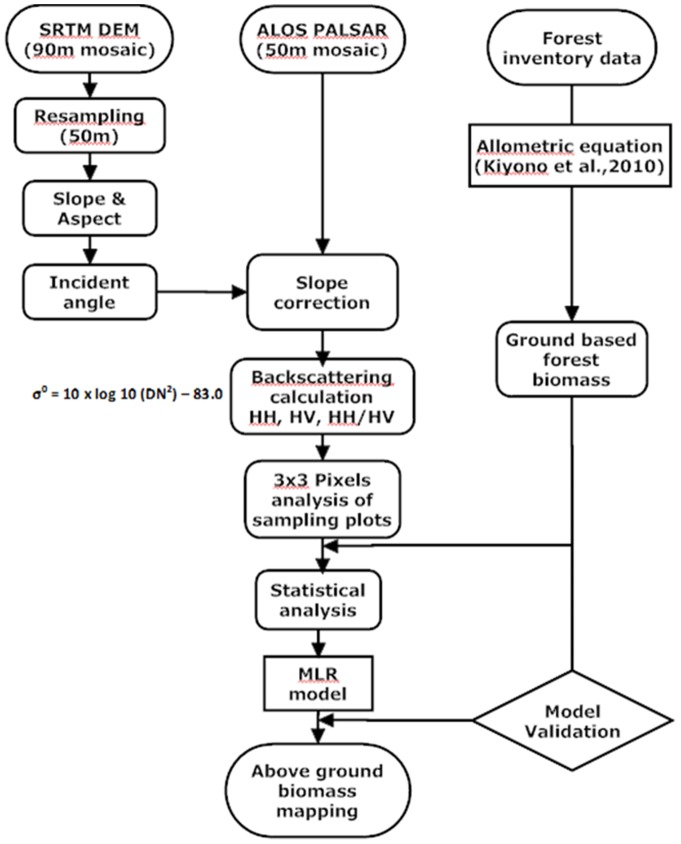
Flow chart of the methodology.

Multi-linear regression (MLR) analysis using the stepwise forward method was conducted relating the σ^o^ of PALSAR to the corresponding field calculated biomass. It was used to analyzed the relationship between the dependent variable (field measured forest biomass) and the independent variables (PALSAR σ^o^). The size of the sampling window was 3×3 pixels. We calculated average value of 3×3 pixels of PALSAR data around the field based sampling points to minimize spatial variability and satisfy the normal distribution based on spatial homogeneity. MLR model were developed based on field measured biomass and PALSAR backscatter. This MLR model was applied to the PALSAR 50 m mosaic data to estimate the biomass of all Cambodia. Finally validation was used to evaluate the accuracy of the model by comparing PALSAR estimated AGB to the field derived AGB. The detailed methodology is shown in the flow chart (figure 3).

## Results and Discussion

**Figure 4:**
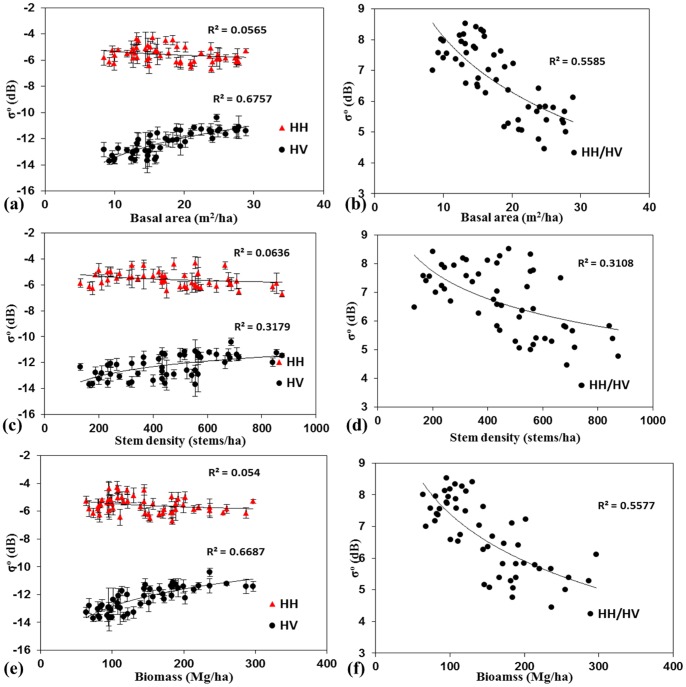
PALSAR 2009 σ^o^ HH, HV and HH/HV plotted against basal area (a, b), stem density (c, d) and biomass (e, f).

σ^o^ HH, HV and HH/HV is plotted against basal area, stem density and biomass respectively. Figure 4 a and b represents the relationship between PALSAR σ^o^ (HH, HV) and (HH/HV) with respect to basal area. Field measured basal area shows a significant relationship with the σ^o^ HV (R^2^ = 0.67) as compared to σ^o^ HH (R^2^ = 0.05). Figure 4 c and d represents the relationship between PALSAR σ^o^ (HH, HV) and (HH/HV) with respect to stem density. Field measured stem density shows the poor relationship with the σ^o^ HV (R^2^ = 0.32) and σ^o^ HH (R^2^ = 0.06) respectively. This is mainly because tree density depends on the forest type, tree species and site conditions. Figure 4d also shows poor relationship between σ° HH/HV with tree density (R^2^ = 0.3). Figure 4e shows the relationship between PALSAR σ^o^ (HH) and (HV) with field estimated biomass. Field measured biomass shows a significant relationship with the σ^o^ HV (R^2^ = 0.67) as compared to σ^o^ HH (R^2^ = 0.05). High σ^o^ HH in low biomass region was noticed because of the high surface scattering from the plots covered by dry leaves and grasses, which increases the surface roughness. The reason why, σ^o^ HV polarization produces better correlation than σ^o^ HH is because of the volume scattering in forest areas enhances the cross-polarization returns with the increase in biomass. σ^o^ HV is less influenced by soil and vegetation moisture than σ^o^ HH [64]. VanZyl (1993) [65] also noticed that HV is less influenced by topography. Other studies also reveal that the σ^o^ HV is more sensitive to forest biomass compared to σ^o^ HH [66], [67], [30].

We have observed different backscattering properties from the same biomass region (Figure 4e at biomass 100–150 Mg/ha) because of the difference in canopy and their distribution. Evergreen forests with multi-story tree structure shows high backscattering as compared to deciduous forests of the same biomass class. A loss in sensitivity of PALSAR signal appeared to occur at approximately 150–200 Mg/ha biomass (Figure 4e). Figure 4f shows the strong relationship between PALSAR σ° HH/HV with biomass (R^2^ = 0.56). Therefore, polarization ratio is a useful parameter for biomass estimation. These results have a higher saturation point and less noise as compared to previous studies using PALSAR HV data [27], [30]. A similar saturation point using PALSAR HV data was noticed by Mitchard et al., (2011) [35] in the Savanna forest of central Africa. We may have achieved this range of saturation and more accurate results compared to previous studies because of our access to good quality, well geo-coded forest inventory data for a relatively flat area.

The MLR Model for biomass estimation was developed using σ^o^ HV and σ^o^ HH/HV because HV and HH/HV shows strong correlation with biomass. σ^o^ HH data was not used for regression modelling, since its dependence on biomass was weak. The resulting regression model is given in equation 2. σ^o^ HV is dominated by volume scattering from woody elements of trees, so that HV is strongly related to AGB [68]. For the HH polarization, ground conditions can affect the biomass backscattering relationship, because HH backscatter comes mainly from trunk-ground scattering [69].
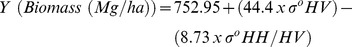
(2)where σ^o^ is backscattering coefficient in dB for different polarization.

**Figure 5:**
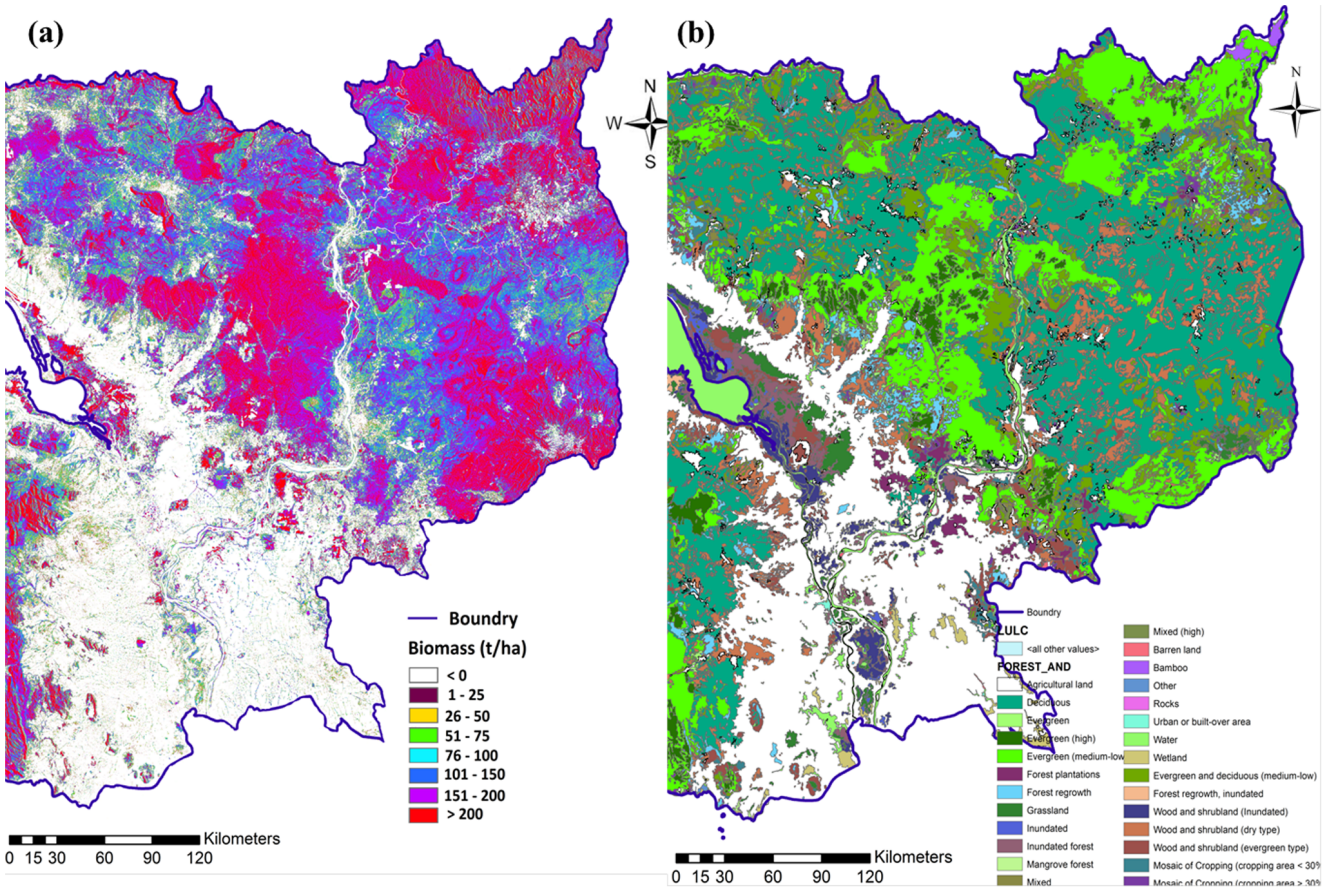
PALSAR derived AGB (Mg/ha) map of Cambodia (a) LULC map of the area (b).

The MLR model was applied to the PALSAR 50 m mosaic data to generate a national level biomass map. Figure 5a shows the biomass map of the Cambodia. The biomass values were classified into 8 classes. The deforested area shows a zero biomass value. Figure 5b shows the land use land cover (LULC) map of the same biomass region. Comparing the biomass map (Figure 5a) with the LULC map (Figure 5b) shows the high biomass region (>200 Mg/ha) mostly falling into the evergreen high and medium low class of the LULC map. However, in the mountainous area (northern part) the biomass map shows variation because of topographic effects. The low biomass region (150–200 Mg/ha) was mostly found in the mixed forest type and the lowest biomass region (50–150 Mg/ha) was mostly found in deciduous forests. The results from this study are preliminary, but it shows the potential of freely available PALSAR 50 m mosaic data.

**Figure 6:**
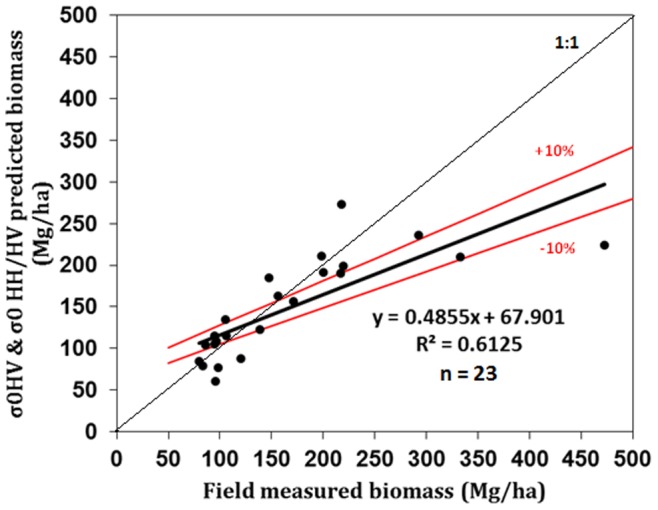
Relationship between PALSAR predicted biomass plotted against field measured biomass.

Figure 6 shows the validation results of PALSAR derived biomass. The accuracy of PALSAR predicted AGB decreases as the biomass increases because of the saturation of PALSAR signal. It shows a significant coefficient of correlation R^2^ = 0.61. The overall root mean square error (RMSE) for this data is 63 Mg/ha; however RMSE decreases to 19 Mg/ha if only values below 100 Mg/ha are considered or down to 21 Mg/ha for values up to 200 Mg/ha. The high variation in errors are present in the high biomass region i.e. >200 Mg/ha. We have predicted two types of uncertainties a) calculation uncertainty of biomass from field data using allometric equation because of the lack of species-specific allometry, small plot sizes and the exclusion of small trees (<10 cm) DBH and b) saturation of PALSAR signal at high biomass regions and topographic effects.

**Figure 7:**
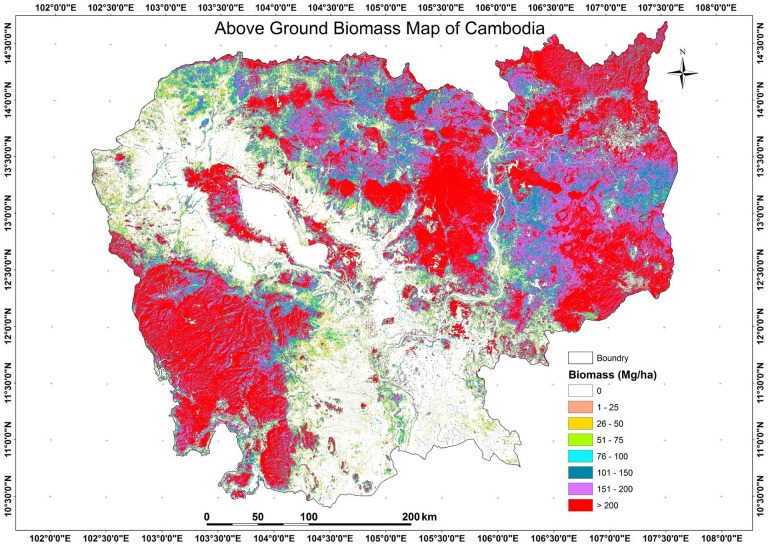
Cambodian AGB map based on PALSAR 50

**Figure 8:**
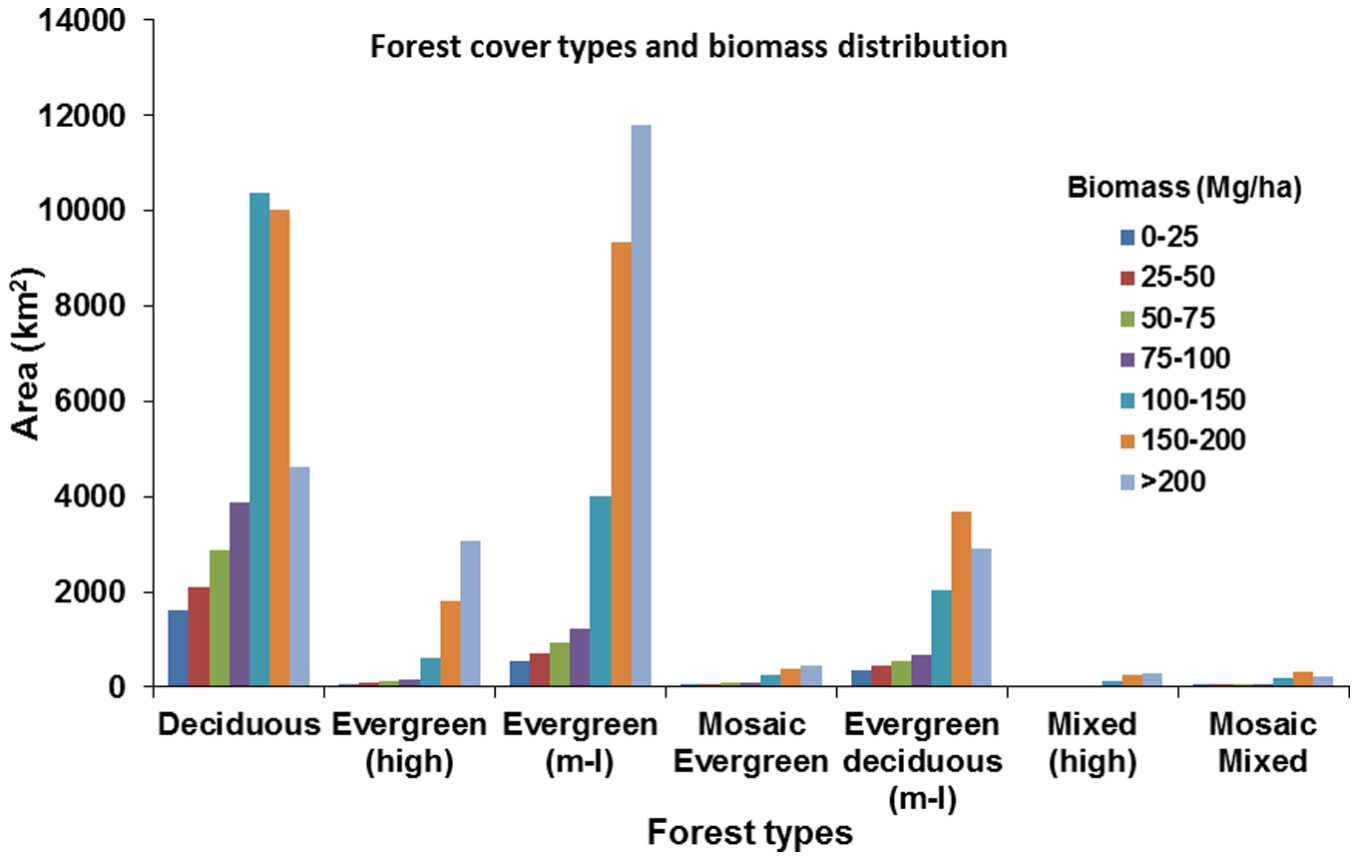
Biomass distributions with forest cover types of Cambodia.

Figure 7 illustrates the national level AGB distribution, which indicates high heterogeneity in AGB class. The high AGB region is mainly found in Ratnakiri, Mondolkiri, Kampong Thom and Koh Kong province of Cambodia. Figure 8 summarizes the distribution of AGB in various types of forests of Cambodia. Evergreen forests have the highest AGB in the >200 Mg/ha class, whereas deciduous forests have in the 100–150 Mg/ha class. Cumulatively, the largest AGB stock is in evergreen forests followed by deciduous forests. The high AGB of evergreen dense forests may be associated with good environmental factors such as relatively better water, soil and temperature conditions and less intensity of human activities. Some sites in evergreen forests of Kampong Thom province with deforestation may be because of high human activity in the flat area with high AGB forest. The majority of Cambodia's forests fall in a range of 100–200 Mg/ha (∼52%) and only 20% of total forest area were <100 Mg/ha. About 28% of the total Cambodia's forests have a value >200 Mg/ha, which is highly significant from the climate change mitigation point of view. Conservation and management of these high biomass forests should be a high priority for increasing the carbon stock as well as for biodiversity conservation.

We also compared our results with Sato, (2011) [70] who estimated biomass values generated based on 100 permanent sampling plots (PSPs) in evergreen and deciduous forests of Cambodia. Table 3 shows the comparison of forest carbon stock in Cambodia based on PALSAR 50 m mosaic data and PSPs based biomass estimation. PALSAR estimated value of total AGB of evergreen forests to be about 347±104 Tg-C, which is close to values estimated by PSPs method. PALSAR estimated value of carbon stock in deciduous forests is about 238±71 Tg-C, which is also close to the PSPs estimated carbon stock value. Therefore we could say that the PALSAR 50 m mosaic generated biomass is reasonable and can be used for further studies. However, a more accurate biomass map is really needed for more accurate climate change modelling.

**Table 3:** Comparison of forest carbon stock in Cambodia based on PALSAR 50

Forest carbon stock in Cambodia based on PALSAR 50 m mosaic data		
Forest types	Forest area (km^2^)	Total Carbon stock (Tg-C)
Evergreen forest	36,140.3	347.42±104.2 (with 30% uncertainties)
Deciduous forest (It does not include mosaic deciduous forest)	35,729.6	238.71±71.6 (with 30% uncertainties)
Mixed forest	12,588.7	102.66±30.8 (with 30% uncertainties)
**Forest carbon stock in Cambodia estimated by Sato T., 2011 based on 100 PSPs**		
Evergreen forest	36,689	467.2±291.5
Deciduous forest	46,921	158.2±110.8

The national level biomass map (Figure 7) will assist Cambodian forestry administration, land managers, policy makers and civil society to become better informed about the likely result of their policies and program in reducing national GHG emissions from land use change. The biomass map could also be used as an additional tool for forest conservation and forest management strategies of Cambodian government.

## Conclusion

Biomass information is useful for calculation of amount of carbon loss due to deforestation activity. In this study, a method for estimating national level biomass map using PALSAR 50 m mosaic data has been developed and evaluated. In this study correlation analysis was used to assess the relationship of AGB and other forest biophysical parameter measured from field data with PALSAR 50 m FBD data. σ^o^ HV and HH/HV shows good correlation with forest biomass. A multi-linear regression model approach was used to predict the biomass using field based measurement and PALSAR backscattering. Our results showed that most of the Cambodian forest (52%) falls into the 100–200 Mg/ha biomass value. About, 28% of Cambodian forest falls into biomass class >200 Mg/ha. The total biomass in evergreen and deciduous forests show good synergies with 100 PSPs estimated biomass, although the methodological approaches are different. Such a national level biomass map is not very precise and accurate but it can provide general information about biomass distribution which is needed for forest management practices in a cost effective way. PALSAR 50 m mosaic data shows saturation at about 150–200 Mg/ha. The saturation problem of SAR data can be overcome using polarimetric-interferometry SAR (PolInSAR) technique or P-band SAR data. For more precise estimation we must look forward for the P-band SAR or DESDynl satellite system in the future.
